# Controversies in the Management of AML in Older Patients: A Canadian Perspective

**DOI:** 10.3390/curroncol33070431

**Published:** 2026-07-18

**Authors:** Andre C. Schuh, Joseph Brandwein, Mahmoud Elsawy, David Sanford, Brian Leber

**Affiliations:** 1 Departments of Medical Oncology and Hematology, Princess Margaret Cancer Centre, University Health Network, Toronto, ON M5G 2M9, Canada; 2 Department of Medicine, University of Toronto, Toronto ON M5S 1A8, Canada; 3 Division of Hematology, Department of Medicine, University of Alberta, Edmonton, AB T6G 2G3, Canada; 4 Division of Hematology and Hematologic Oncology, Department of Medicine, Dalhousie University, Halifax, NS B3H 2Y9, Canada; 5 QEII Health Sciences Centre, Halifax, NS B3H 3A7, Canada; 6 Division of Hematology, University of British Columbia, Vancouver, BC V5Z 1M9, Canada; 7 Department of Medicine, McMaster University, Hamilton, ON L8S 4L8, Canada

**Keywords:** acute myeloid leukemia, elderly patients, genetically defined risk, less-intensive treatment, risk stratification

## Abstract

In the companion article in this issue of *Current Oncology*, ‘Management of AML in Older Patients: An Updated Canadian Consensus’, the authors have presented the third iteration of Canadian consensus guidelines on AML treatment in the elderly. While many aspects of AML treatment in the elderly have become better defined, some old questions remain and new questions and controversies have arisen. Here, we address three topics on which there is, at this time, no universal consensus. The first is the development and features of new risk-stratification systems specifically aimed at older, less-intensively treated patients. Several different systems now exist in parallel, potentially causing confusion amongst clinicians. The second topic is good-prognosis AML subtypes (*IDH1-* and *NPM1*-mutated AML). In the Canadian context, *IDH1*-mutated AML is of particular interest due to the new availability of ivosidenib in Canada. The third topic is adverse-risk AML subtypes (*FLT3-* and *TP53*-mutated AML). *FLT3*-mutated AML remains problematic, although it is anticipated that new drug approvals and measurable residual disease-based treatment approaches may alleviate this, at least in part. And in particular, *TP53*-mutated AML remains a major problem. Ongoing clinical trial enrollment is essential.

## Simple Summary

Non-intensive AML treatments for older/frail patients have evolved considerably over the last decade, with an improved understanding of AML disease biology. This has led to a better understanding of risk in AML, and within the past two years, several different ‘risk-stratification’ systems have been developed. These systems enable clinicians to assess risk in individual patients, which in turn allows them to better define effective treatment approaches for different subtypes of AML. For example, there are several subgroups of *NPM1*-mutated AMLs with distinct risk types that may define specific treatment approaches. *FLT3*-mutated AML remains problematic, although it is anticipated that new drug approvals and new treatment approaches may alleviate this, at least in part.

## 1. Introduction

In the companion article in this issue of *Current Oncology*, ‘Management of AML in Older Patients: An Updated Canadian Consensus’, the authors have presented the third iteration of Canadian consensus guidelines on acute myeloid leukemia (AML) treatment in the elderly. While many aspects of AML treatment in the elderly have become better defined and, in some cases, almost formulaic—albeit progressively more complicated—over time, and with better outcomes, some old questions remain and new questions and controversies have arisen, particularly in the Canadian context. Here, we address in light of emerging data, several topics for which universal consensus does not yet exist. The first is the advent of new risk-stratification systems.

With the expanded use of less-intensive treatment approaches in older patients, it has become clear that conventional risk stratification approaches are inadequate in this setting. As a result, the last few years have seen an explosion of risk-stratification models aimed specifically at older, less-intensively treated patients, with iterative model refinements often following one another in short order. As a result, there now exist several systems used in parallel, raising the risk of confused clinician understanding and communication. Here, we put these newer scoring systems into historical context and compare/contrast their individual features, aiming to provide clarity.

Next, we discuss two good-prognosis AML subtypes—*IDH1*- and *NPM1*-mutated AML—in greater detail. *IDH1*-mutated AML is instructive due to the new availability of ivosidenib in Canada. Important questions include the distinct nature of ivosidenib-related differentiation syndrome (DS) and the impact of ivosidenib availability on the turn-around-time requirements for the diagnostic molecular testing needed for optimal use of the drug. Finally, *IDH1*-mutated AML also hints at the potential importance of co-mutational status on defining optimal drug choice.

*NPM1*-mutated AML is then discussed in the context of the classification systems outlined above, adding biological nuance to the scoring systems discussed earlier. *NPM1*-mutated AML is not a homogenous entity. Rather, there exist several subtypes of *NPM1*-mutated AML that, taken together, underscore and help clarify the intimate relationship between genetic and phenotypic risk stratification and drug sensitivity.

Finally, two adverse-risk AMLs—fms-like tyrosine kinase 3 *(FLT3)*-mutated and *TP53*-mutated—are discussed in the context of initial therapy and the potential for maintenance therapy and allogeneic stem cell transplantation (alloSCT). FLT3 inhibitors are topical due to:

- Their place in evolving risk-assessment systems;
- Drug choice questions arising from the new availability of multiple FLT3 inhibitors in Canada;
- An expansion of approved drug indications;
- A greater emphasis on measurable residual disease (MRD)-based alloSCT decision-making;
- A better understanding of maintenance therapy both after consolidation chemotherapy and after alloSCT.

*TP53*-mutated AML is discussed as an ongoing treatment challenge with limited and unsatisfactory drug and alloSCT treatment options, with the goal of clarifying potentially confusing treatment decisions. *TP53*-mutated AML treatment remains a major unmet need. Ongoing clinical trial enrolment is essential.

### Methods

In parallel with the development of the updated consensus statement, a panel of Canadian Leukemia Study Group/Groupe canadien d’étude sur la leucémie (CLSG/GCEL) members with expertise in the clinical management of AML was convened to develop this document. As per the consensus statement, the panel convened to identify key issues with respect to the management of AML in older patients. Relevant literature was identified by panel members, who also provided insights based on personal and institutional experience and judgment. No formal evaluation of evidence was conducted.

Panel members then worked individually and in smaller groups to develop outlines, as well as to draft sections of the document. These contributions were combined into a document that was reviewed by the whole panel, with an opportunity for dissenting opinions to be noted.

The CLSG/GCEL will post this document on its website (https://clsg.ca) following publication, with the intention of iteratively updating sections of the document as new data become available. AML management is a rapidly evolving area. Online updates by section will allow these recommendations to remain as current as possible over time.

## 2. Risk Stratification in Patients Receiving Less-Intensive Therapy

A central issue in AML management today is the risk stratification of AML patients receiving less intensive therapy. Early AML risk stratification strategies, such as that developed in the UK Medical Research Council (MRC) AML 10 trial, were strictly cytogenetic [1]. MRC AML 10 investigators defined three cytogenetic risk groups (favorable, intermediate and adverse) that, for many years, helped support treatment vs. no treatment and transplant vs. no transplant decision-making. Over 10 years later, with improved understanding of the molecular pathogenesis of AML and the increased availability of molecular testing, the 2010 European LeukemiaNet (ELN) recommendations added defined molecular abnormalities to the prior cytogenetic schema to define genetic risk in AML [2]. This approach became progressively more comprehensive over the course of the ELN 2017 and ELN 2022 recommendations [3,4].

However, these historical risk stratification systems became less predictive over time. They were based on data from younger patients (age ≤ 55/60) treated with intensive chemotherapy (commonly the 7 + 3 regimen or a variant thereof). They also predated gemtuzumab ozogamicin and *FLT3* inhibitors, less intensive therapy approaches (e.g., hypomethylating agent (HMA) monotherapy, HMA + venetoclax, HMA + ivosidenib) and maintenance therapy with oral azacitidine, as well as the more widespread availability of alloSCT. Considering that the utility of a risk stratification system is defined by the combination of the patient population included and the treatment received, it was unlikely that the conventional risk-assessment approach could be used to predict response and support treatment decisions in older patients unfit to receive intensive chemotherapy. Alternative risk assessment strategies were thus needed in this older patient group. Multiple groups have therefore tackled risk assessment in older AML patients receiving less intensive therapy.

AML risk can be defined by both genetic and phenotypic criteria. It seems obvious that AML genetics and phenotype are linked, although the relationship between the two is only recently being elucidated. Most approaches to risk stratification in the less-intensively treated patient with AML have focused on genetically defined risk. Here, we will focus primarily on genetically defined risk but will thereafter return to phenotypic approaches to risk assessment.

### 2.1. Genetically Defined Risk

#### 2.1.1. VIALE-A Study and mPRS (4-Gene Classifier)

The phase III VIALE-A trial compared azacitidine + venetoclax with azacitidine + placebo in previously untreated AML patients who were ineligible for intensive chemotherapy due to age ≥ 75 years or due to the presence of specific predefined comorbidities [5]. Response and survival data are shown in Table 1. Median overall survival (OS), complete remission (CR), and composite complete remission (CR/CRi (CR with incomplete hematologic recovery) were all significantly better with azacitidine + venetoclax than with azacitidine alone. Results also showed that in patients with *IDH1* or *IDH2* mutations, the CR/CRi rate was significantly higher with azacitidine + venetoclax (75.4% vs. 10.7%). This was a novel observation at the time, and suggested that *IDH* mutations might be an important prognostic factor [5].

A pooled analysis of the VIALE-A trial [5] taken together with the results of an earlier phase 1b study [6] examined prognostic stratification in azacitidine + venetoclax-treated patients according to the 2017 and 2022 ELN risk classifications [7]. Table 2 shows that patients treated with azacitidine + venetoclax had higher response rates and longer median survival than those treated with azacitidine alone across all risk categories defined by ELN 2017 [3] and ELN 2022 [4].

Notably, the relative ratios of patients in each risk category differed between ELN 2017 [3] and ELN 2022 [4] classifications. And most importantly, when only the patients who received azacitidine + venetoclax were considered, overall survival was similar for patients with favorable and intermediate risk when defined by ELN 2017 [3], and for patients with intermediate- and adverse-risk when defined by ELN 2022 [4]. Thus, as expected, neither of these risk classifications (both based largely on outcomes in younger/fitter patients treated with intensive chemotherapy as noted above) discriminated well among the three ELN risk categories, providing only limited prognostic value.

The azacitidine + venetoclax-treated patients in this pooled analysis were subsequently evaluated further to determine the potential prognostic significance for OS of over 30 cytogenetic aberrations and molecular abnormalities. A new risk model, the Molecular Prognostic Risk Signature or mPRS (also referred to as the 4-gene classifier) was subsequently developed based on three prognostic risk signatures defined by the mutational status of only four genes (Table 3) [7]. When applied in an analysis of all VIALE-A patients who received azacitidine + venetoclax, the mPRS risk model clearly differentiated this population into three distinct survival groups—higher, intermediate, and lower benefit—with median OS of 26.5, 12.1 and 5.5 months, respectively. Although impressive response rates were previously reported in the original VIALE-A publication for patients whose leukemia carried either *IDH1/2* or nucleophosmin (*NPM1*) mutations, neither mutation was found to be predictive of OS in the mPRS analysis, and thus these mutations were not included in the risk model.

Subsequent refinements to the mPRS model followed three main approaches, taken alone or in combination:

- Repeat mPRS-type analysis with additional patient cohorts;
- Analysis of patients treated with alternative less-intensive protocols (i.e., non venetoclax + HMA);
- Inclusion of additional genes not included in the original VIALE-A study.

#### 2.1.2. MDACC mPRS Validation

In the first follow-up to the original mPRS report [7], investigators at the MD Anderson Cancer Center (MDACC) evaluated retrospectively the outcomes of 159 patients who had been treated at their center with HMA (azacitidine or decitabine) + venetoclax [8]. They observed a similar lack of discrimination among patient groups by ELN 2017 [3] and ELN 2022 [4] criteria and validated the mPRS risk model in their cohort. Importantly, they demonstrated further that the mPRS score was prognostic not only for azacitidine + venetoclax, but also for the decitabine + venetoclax combination.

#### 2.1.3. Mayo Clinic Genetic Risk Model

The mPRS approach was further refined in a Mayo Clinic study [9]. In an analysis of 400 adult patients with newly diagnosed AML treated with HMA + venetoclax, the investigators identified genetic factors associated with better vs. inferior initial response, as well as with longer vs. shorter OS (Table 4, section A). With respect to OS, the former included *IDH1* and *IDH2* mutations, and the latter included adverse karyotype by ELN 2022, *KMT2A* rearrangements (*KMT2Ar*), and *TP53* and *KRAS* mutations. They then developed a points-based scoring system for OS, with patients divided into three risk groups: low, intermediate, and high (Table 4, section B).

When compared to the mPRS approach, the Mayo Clinic model discriminated better between low- and intermediate-risk and between intermediate- and high-risk groups. The Mayo Clinic model was also better able to predict 3-year survival. Importantly, the Mayo Clinic model improved upon the mPRS system by incorporating additional genetic factors (e.g., ELN 2022 adverse-risk karyotype, *KMT2A* rearrangements). The Mayo Clinic study also analyzed a wider range of genes than were assayed in VIALE-A. For example, mutations in *DDX41* (not included in VIALE-A) were associated with an improved CR/CRi rate.

#### 2.1.4. Beat-AML 2024

Notably, the above studies all analyzed patients treated exclusively with HMA + venetoclax. Whether these risk-stratification approaches would apply similarly to patients treated with alternative, less-intensive drug combinations was not known. In parallel, therefore, other studies analyzed alternatively treated patients.

The Beat-AML consortium in the United States developed a different approach to overcoming the shortcomings of the ELN 2022 risk classification system when applied to patients treated with less-intensive therapy: Beat-AML 2024 [10]. The investigators grouped ELN 2022 favorable and intermediate-risk patients into a newly defined ‘Beat-AML favorable risk’ group. They then refined the ELN 2022 high-risk group using a mutation score that incorporated independently significant gene mutations (*IDH2*, *MLL2*, *KRAS* and *TP53*) in high-risk patients, thereby creating new high- and intermediate-risk groups. This analysis more precisely defined adverse risk status and provided additional prognostic information: the reported survival curves clearly discriminated among the three new patient risk groups. However, only about one-third of the patients had received HMA + venetoclax. The remaining patients were treated with other combinations or single agents (HMA alone, 5%; HMA combinations (no venetoclax), 54%; venetoclax combinations (no HMA), 4.2%; others, 1.8%), raising questions about the generalizability of this predictive score in clinical practice.

#### 2.1.5. AGILE Study

Also diverging from the HMA + venetoclax combination, the phase III AGILE study compared azacitidine + ivosidenib to azacitidine + placebo in patients with previously untreated *IDH1*-mutated AML who were ineligible for intensive chemotherapy based on criteria similar to those of VIALE-A [11]. Response rates were higher and OS was longer with azacitidine + ivosidenib than with azacitidine alone (Table 5). The OS of the azacitidine + ivosidenib arm in AGILE (29.3 months) compared favorably with the OS of *IDH1*-mutated patients in the VIALE-A study who were treated with azacitidine + venetoclax (10.2–15.2 months) [11,12]. Thus, while in VIALE-A, venetoclax treatment in patients with *IDH1* mutations was associated with high CR/CRi rates but not with longer OS, the use of ivosidenib in the AGILE study did lead to longer OS, underscoring that the mPRS approach had little predictive value in patients not treated with HMA + venetoclax.

#### 2.1.6. ASTRAL-1 Study

In parallel with the above analyses, a number of other studies revealed that additional mutations not analyzed in VIALE-A also have a potential prognostic role in less-intensively treated AML. In particular, several publications have shown that in AML patients, *DDX41* mutations are associated with favorable OS outcomes. Jahn et al. carried out targeted DNA sequencing on 263 genes in 604 patients enrolled in the ASTRAL-1 trial (the largest study to date of older AML patients receiving less intensive therapy, with >800 patients enrolled) [14,15]. Again, overall survival curves according to the ELN 2022 risk-classification system did not discriminate well among favorable-, intermediate-, and adverse-risk patients. However, when new risk groups were defined on the basis of mutational and cytogenetic features, survival curves clearly differentiated between patients with *DDX41* mutations vs. patients with *FLT3*-ITD and *TP53* mutations. A forest plot of the impact of mutational and cytogenetic status on OS also identified *DDX41* mutations as a highly favorable prognostic marker, and *FLT3*-ITD and (particularly) *TP53* mutations as unfavorable prognostic markers [14], adding to the prognostic models described above.

#### 2.1.7. ELN 2024 Genetic Risk Classification

In light of the disparate and somewhat confusing prognostication data described above, when taken together with the prior ELN 2017 [3] and ELN 2022 [4] classifications, the new ELN 2024 recommendations on genetic risk classification of adults with AML receiving less intensive therapies (Table 6) were devised to better reflect current practice by integrating a range of treatment approaches with a wider range of genetic abnormalities than had been assessed in VIALE-A [16]. The ELN 2024 recommendations closely resemble the mPRS approach but with the addition of *DDX41* mutations as a good risk feature, as well as the stipulation that *IDH1* mutations are good-risk only if ivosidenib is used.

The ELN 2024 recommendations were a clear step forward [16], but they also raised some questions regarding their comprehensiveness, including:

- Should AMLs with mutations particularly sensitive to venetoclax (e.g., *NPM1*, *DDX41*, or *IDH1/2*) and without co-occurring signaling mutations (such patients may have unusually long OS) encompass a separate subgroup?
- Are there other favorable-risk markers similar to *NPM1*, *DDX41*, or *IDH1/2*?
- Are *NRAS* and *KRAS* mutations truly similar with respect to risk?
- Do other *RAS*/receptor tyrosine kinase pathway mutations (i.e., *CBL*, *NF1*, and *PTPN11*) similarly affect prognosis?

#### 2.1.8. Refined ELN 2024 Risk Stratification

Very shortly after the publication of the ELN 2024 recommendations, a refinement was proposed that addressed some of these questions. The ‘refined’ ELN 2024 risk stratification system [23] was based on a retrospective analysis of 279 patients in three U.S. centers who were treated with frontline HMA + venetoclax combinations. These patients were younger than the VIALE-A population (median age 72 years and 62% aged ≥ 70 years), and 17% went on to alloSCT. Median OS was 11.4 months (median follow-up 28 months; 95% CI 9.0–14.4). This was shorter than the 14.7 months median OS in VIALE-A, but was similar to that observed in other retrospective cohorts. The ‘refined’ ELN 2024 approach was subsequently validated in an additional 430 patients treated with HMA + venetoclax combinations in UK NHS hospitals.

When the ELN 2024 risk stratification system was applied to this patient population, the results (Table 7) suggested that [16,23]:

- Favorable-risk patients with *NPM1*, *IDH1/2* and *DDX41* mutations had particularly long survival, whereas others bearing mutations also considered favorable, but without these three mutations, had median survival comparable to patients classified as intermediate risk by ELN 2024.
- When identified at diagnosis, *KRAS* (but not *NRAS*) and *PTPN11* mutations were prognostic for poor OS. Both the mPRS scoring system and ELN 2024 considered *KRAS* and *NRAS* mutations to be equivalent; neither system considered *PTPN11*.

After accounting for potential differences in prognostic weight among genetic abnormalities and the non-mutually exclusiveness of mutations, and after adjusting for alloSCT, the ELN 2024 [16] risk groups could be re-classified by including mutations that remained significant after multivariate analysis [23]:

- Favorable: mutated *NPM1*, *IDH1/2*, *DDX41* (with wild-type *KRAS*, *NRAS*, *PTPN11*, *FLT3-ITD*, *TP53*).
- Intermediate: mutated *FLT3*-ITD, *NRAS*, other mutations not classified (with wild-type *KRAS*, *PTPN11*, *TP53*).
- Adverse: mutated *KRAS*, *PTPN11*, *TP53*.

The effect of these mutations on the more precise definition of patient risk within ELN 2024 categories was striking (Table 7), with the revised system defining better OS potential more precisely (Table 8). Overall, ‘refined’ approaches such as the ‘refined ELN 2024’ should therefore permit more precise prognostication and enhanced treatment individualization.

#### 2.1.9. PRISM (Prognostic Risk Integration for Survival Modeling) Study

While the scoring systems outlined above have followed a trajectory of iterative improvements that have enhanced prognostic precision and treatment individualization, all have shortcomings that must be overcome to optimize treatment for a larger proportion of patients. Such refinements are found most definitively in the recently described PRISM model, which surpasses previous approaches in its comprehensiveness [24]. To date, it has been presented only in abstract form, limiting the ability to draw firm conclusions.

The goal of the PRISM study was to assess a patient cohort that was large enough to accurately analyze the prognostic significance, both singly and in combination, of a comprehensive number of mutations, as well as of a variety of clinical factors such as secondary AML (sAML) and alloSCT. The PRISM investigators comprised a multisite, multinational consortium that together enrolled 2301 patients with median age of 74 years (training cohort, 1385; internal validation cohort, 587; external validation cohort, 329), of which >97% received HMA + venetoclax (a few patients received low-dose cytarabine (LDAC) + venetoclax). The training and internal validation cohorts (a 70:30 split of internal patients) were stratified by OS events, alloSCT realization, and *TP53* status.

Thirty AML-related mutations were scored, together with other biological and patient features including ELN 2022 favorable-, intermediate-, and adverse-risk cytogenetics; AML type (*de novo*, sAML, and tAML); and age at diagnosis. Next, variables were identified with ≥75% unidirectional effects (i.e., favorable or adverse) on OS, and a model was developed that contained seven variables associated with a more favorable outcome (OS HR < 1) and ten variables associated with a more adverse outcome (OS HR > 1), with each variable assigned a specific weight (Table 9). Patient-specific scores were calculated as the weighted sum of variables present and overall patient scores were divided into tertiles, resulting in three score-based risk groups (low-, moderate-, and high-risk), with the groups enriched as expected for specific genetic/clinical features.

Notably, the PRISM score resulted in the redistribution of >50% of patients as classified by the mPRS approach (Table 10). In addition, compared to the mPRS approach, OS stratification was improved considerably with the PRISM prognostication approach, with better discrimination between low-risk and both moderate- and high-risk groups (Table 11).

The PRISM score relies on specific prognostic weights of co-occurring genetic mutations, rather than just scoring them in a yes/no manner. It also includes several clinical variables such as age, sex, and sAML that also have prognostic importance. As a result, the PRISM score likely represents the most comprehensive and precise prognostication system to date for assessing survival in older patients treated with HMA + venetoclax. As this risk score may be difficult to calculate, the authors have provided a web application that determines both the PRISM and mPRS risk scores in parallel (prism-aml.com).

### 2.2. Phenotypically Defined Risk

As illustrated above, investigations into risk assessment in older AML patients receiving less intensive therapy have to date focused mostly on genetically defined risk [25]. However, AML cell phenotype has long been known to play a role in venetoclax sensitivity/resistance. Specifically, monocytic differentiation has been associated with venetoclax failure [26,27]. Two recent publications have helped clarify this phenotypically defined risk [28,29]. Venetoclax sensitivity is influenced by the leukemic stem cell (LSC) differentiation state. More differentiated cells are more monocytic, less venetoclax-responsive and, importantly, have lower levels of BCL2 and higher levels of MCL1. Waclawiczek et al. developed a ‘mediators of apoptosis combinatorial score’ (MAC score) that links the ratio of intracellular protein expression of BCL2, BCL-xL and MCL1 in LSCs [28], thereby quantifying the likelihood of venetoclax response. The MAC score is flow cytometry-based and can predict individual patient response as well as potentially provide information on the optimal duration of azacitidine + venetoclax therapy. The MAC score is currently not used widely in clinical practice.

Lachowiez et al. subsequently applied the MAC score concept in a large retrospective study of AML patients treated with HMA + venetoclax [29]. Results showed that in certain AML subgroups (particularly *NPM1^wt^*), a monocytic phenotype is associated with decreased overall survival following venetoclax-based therapy. The study also confirmed that differentiation state and venetoclax sensitivity are defined largely by genetics, as per the mPRS, ELN 2024 and refined ELN 2024 risk models [7,16,23]. These observations underscore the complex interplay of genetics, AML differentiation state, and antiapoptotic protein expression, and suggest that analysis of leukemic cell phenotype, particularly if it is doable with a simple assay, might provide an avenue to improving AML stratification and prognosis.

## 3. Treatment of Older AML Patients with Good-Risk Mutations: IDH1 and NPM1

### 3.1. IDH1-Mutated AML

*IDH1/2* mutations have been described for over fifteen years, and the elucidation of their effects has contributed significantly to our understanding of AML development and biology. *IDH1* and *IDH2* encode the cytoplasmic and mitochondrial isoforms, respectively, of the citric acid cycle enzyme isocitrate dehydrogenase (IDH). Wild-type *IDH* converts isocitrate to α-ketoglutarate (α-KG), a cofactor for multiple α-KG-dependent dioxygenases including TET2 (a DNA demethylase) and the JmjC family of histone demethylases, thereby promoting normal epigenetic regulation of hematopoiesis. The pathogenic mutations in *IDH1/2* confer neomorphic gain-of-function activity, converting α-KG to the oncometabolite 2-hydroxyglutarate (2-HG). The resultant depletion of α-KG, together with the accumulation of 2-HG (a competitive inhibitor of α-KG-dependent dioxygenases), leads to profound epigenetic dysregulation, impaired differentiation of hematopoietic stem and progenitor cells, aberrant proliferation, and leukemogenesis [30,31,32].

*IDH1* mutations are found in approximately 6–10% of AMLs overall, with a higher incidence in normal karyotype AML and with increasing age [32,33,34]. *IDH1* mutations can occur relatively early in leukemogenesis and thus can be more stable between first diagnosis and relapse than later mutations such as *FLT3*-ITD [35,36]. With respect to outcome, when considered on their own, *IDH1* mutations are generally associated with inferior outcomes, although their influence is controversial [37,38,39], and can be co-mutation specific [40]. While relatively infrequent compared to some other well-characterized mutations in AML, rarity alone should not preclude the selection of therapy specifically targeted to the biology of *IDH1* mutations.

Treatment of *IDH1*-mutated AML has been markedly altered by the availability of ivosidenib, an *IDH1*-targeted agent. Ivosidenib was approved by the U.S. Food and Drug Administration (FDA) in 2019 [41] and the European Medicines Agency (EMA) in 2023 [42]. It was included in the September 2025 Canadian Drug Association provisional funding algorithm for adult AML [43].

Ivosidenib demonstrated significant monotherapy activity in relapsed/refractory (R/R) AML [44]—an impressive result for a single-agent, mutation-specific therapy in this context. The AGILE trial [11] subsequently evaluated ivosidenib + azacitidine in newly diagnosed *IDH1*-mutated AML and showed significant clinical benefit in this difficult-to-treat population. It is notable that this trial began prior to the publication and broad acceptance of VIALE-A results [5]. At the time, azacitidine monotherapy was the accepted standard of care. (For more on the AGILE trial and *IDH1*-mutated AML, see Section 3.1.1, *Ivosidenib and Azacitidine,* in the companion article in this issue of *Current Oncology*, entitled ‘Management of AML in Older Patients: An Updated Canadian Consensus’.)

Pharmacodynamically, ivosidenib produced a ≥10-fold reduction in serum 2-HG levels across nearly all patients, regardless of response category [11]. This finding indicates that 2-HG suppression is necessary but not sufficient for clinical response. A distinctive feature of *IDH1* inhibition is the early rise in neutrophil counts due to the release of differentiation blockade, analogous in principle to differentiation effects observed with treatment in acute promyelocytic leukemia (APL). Despite concurrent azacitidine therapy, median neutrophil counts increased rather than decreased during early cycles, in contrast to the cytopenias typically observed with venetoclax-based or azacitidine monotherapy regimens. Interestingly, this early release of maturation block for neutrophils was not evident for platelets, indicating that the myeloid differentiation pathway may be particularly sensitive to 2-HG epigenetic effects, and its reversal by IDH inhibitors.

The mechanism of action of ivosidenib (release of the *IDH1*^mut^-mediated hematopoietic differentiation block) underlies both the early benefits and toxicities of ivosidenib. Limited drug-induced differentiation accounts for the early neutrophil count increases and the resultant decreased rates of febrile neutropenia and infections seen with ivosidenib. Excessive differentiation, in contrast, may manifest as differentiation syndrome (see below). Tumor lysis syndrome—a concern with venetoclax—was not observed in this trial [11].

Differentiation syndrome associated with IDH inhibitors differs from the classic pattern observed in APL treated with arsenic trioxide and ATRA [45,46,47]. In APL, the incidence of DS typically peaks between days 7 and 14 of therapy and declines rapidly thereafter. With ivosidenib-based therapy, the peak occurs later (between days 14 and 21) and the risk declines more gradually. Cases continue to appear late in cycle 1, throughout cycle 2, and even beyond. This reflects the dual mechanism of leukemic clearance: while venetoclax and azacitidine act primarily through apoptosis, IDH inhibition both induces apoptosis and restores differentiation. As leukemic differentiation progresses over time, inflammation and cytokine release may emerge later than expected. Clinicians must thus remain vigilant for delayed presentation of DS and avoid attributing signs/symptoms, such as unexplained fevers, dyspnea, hypotension, or pulmonary infiltrates to infection or disease progression, when they may represent DS. Management mirrors DS management in APL: prompt initiation of dexamethasone is critical, along with supportive care (e.g., oxygen supplementation, diuretics for pulmonary edema, and close monitoring). Simultaneous antimicrobial therapy for possible infection/febrile neutropenia may also be required. The notable difference is the longer time window in which DS may appear, necessitating continued awareness beyond the earliest days of therapy.

Identifying patients eligible for *IDH1*-targeted therapy requires timely molecular profiling. A national survey conducted in Canada in May 2024 [43] revealed that while all institutions included *IDH1*, *IDH2*, and other AML-relevant genes in their next-generation sequencing (NGS) panels, turnaround times were often as long as two to three weeks. Such delays challenge the implementation of frontline *IDH*-targeted therapy. The question posed in 2024 was whether to accelerate results by improving full-panel NGS turnaround, or by adopting rapid single-analyte *IDH1* polymerase chain reaction (PCR) assays. Although single-target assays are operationally flexible, they are more expensive. However, a clear precedent is noteworthy: when midostaurin became available for *FLT3*-ITD/TKD-mutated AML, laboratories nationwide implemented rapid *FLT3* testing within days of AML diagnosis to guide induction therapy. Accelerated biomarker turnaround is feasible when clinically necessary.

Ivosidenib received Notice of Compliance from Health Canada in May 2024. In accordance with recommendations from the ELN in 2022 [4], several provinces have designated *IDH1* as a “rapid biomarker” analogous to *FLT3*. These jurisdictions provide funding to ensure test results are available within three to seven calendar days. The remaining provinces are expected to follow suit, although testing methodologies may vary. (There are no leukemia centres in any of the Canadian territories.) As a result, nationwide access to timely *IDH1* testing is expected to improve significantly. Rapid diagnostics matter, because although AML therapy can sometimes be safely delayed, delays should not be the default.

A large German registry study analyzed intensively treated AML patients stratified by time to treatment [48]. Although unadjusted survival did not differ significantly between long and short times to treatment, the analysis was non-randomized, and most patients were treated within 10 days (and many within 4 days) of diagnosis. A similar pattern was observed in unfit AML patients treated with azacitidine + venetoclax, where treatment typically began within 4 days [49]. These findings show that while delays may be safe for selected individuals (and essential for some with acute but reversible comorbidities such as severe pneumonia), molecular diagnostics should still be expedited, especially when targeted therapy may be superior.

The evolving treatment landscape for *IDH1*-mutated AML requires contextualizing the value of targeted therapy against existing standards such as azacitidine + venetoclax. The VIALE-A trial and its precursor phase 1/2 studies demonstrated that venetoclax-based therapy provides meaningful clinical benefit across multiple genetic subgroups, including those with *IDH1* mutations [5,6]. Updated analyses show that patients with *IDH1*-mutated AML treated with azacitidine + venetoclax achieved a CR/CRi rate of 66.8%, compared with only 29.0% in the azacitidine + placebo arm. Median OS was also markedly improved: 10.2 months with azacitidine + venetoclax vs. 2.2 months with azacitidine + placebo [50].

Cross-trial comparisons must always be approached with caution. However, clinicians often do compare venetoclax-based outcomes with the azacitidine + ivosidenib-based outcomes in the AGILE trial. As previously mentioned, AGILE reported a median OS of 29 months, substantially longer than the 10.2 months with *IDH1*-mutated AML in VIALE-A. However, this was not a pre-planned analysis designed to show the difference between treatments for this subgroup [11,50]. Furthermore, although the eligibility criteria of the two studies were very similar, there were marked differences in outcome between the placebo arms that reflect the differences in patient selection, co-mutation landscape, and baseline characteristics between the two studies. These distinctions reinforce that cross-trial comparisons can be problematic.

Because of the efficacy of both these targeted agents when added to HMAs in *IDH1*-mutated AML, multiple early-phase studies have explored combining ivosidenib, venetoclax, and azacitidine in such patients. Based on the promising results of these early phase studies, the EVOLVE-1 multinational randomized Phase 3 trial (NCT07075016) will compare ivosidenib–azacitidine (the AGILE regimen) against a triplet combination including venetoclax. Event-free survival will serve as the primary endpoint, given expected crossover to the experimental triplet regime at relapse, which would confound overall survival analysis.

### 3.2. NPM1-Mutated AML

*NPM1*-mutated AML, historically considered “favorable risk”, is mechanistically complex with multiple biological implications. This mutation leads to aberrant cytoplasmic localization of the NPM1 protein, but importantly, also creates a novel bipartite histone-binding interface dependent upon *XPO-1* that alters chromatin architecture at key regulatory loci, thereby sustaining expression of *HOXA/HOXB*, *MEIS1*, and *IRX* family genes. This epigenetic dysregulation traps progenitors in a stem-like, undifferentiated state. In addition, *NPM1*-mutated AML is nearly always characterized by additional mutations, frequently *DNMT3A*, *FLT3*-ITD, *RAS*-pathway genes, or *IDH* mutations [51].

Because of the wide heterogeneity of co-mutations, and the fact that *NPM1* as a solitary AML mutation is quite rare, several classification schemes have attempted to subtype *NPM1*-mutated AML. The British AML17/19 framework that examined outcomes after different “strengths” of intensive chemotherapy regimens, incorporated *NPM1* mutation subtype and co-mutational patterns to stratify relapse risk and MRD persistence [52]. The results indicated that *NPM1* mutations accompanied by *DNMT3A*, *IDH1/2*, or other favorable-risk mutations tended to have the highest MRD-negativity rates, whereas those with *FLT3*-ITD-high or *RAS*-pathway mutations had substantially lower MRD clearance rates. This observation is consistent with the functional understanding that *FLT3*-ITD and *RAS*-pathway lesions promote proliferation and resistance to apoptosis induced by a wide range of therapies [53,54].

To understand better the implications of co-mutation patterns in AML, the Harmony Alliance investigators moved beyond the single mutation approach of Pappaemmanuil [55] and used unsupervised combinatorial clustering to subclassify all AMLs by mutation combinations. In this work, presented at EHA 2025, they identified 17 gene clusters composed of several driver mutations with distinct co-mutational patterns: 3 of these were *NPM1*-centric [56]. A third novel epigenetic clustering approach, presented at EHA 2024, identified 18 AML-related states, including four major *NPM1*-related epigenetic states [57]. These findings reinforce two important principles. First, *NPM1*-mutated AML is not a homogeneous entity. Second, epigenetic programs may be as relevant clinically as are mutational categories. Such insights are likely to shape future risk-stratification frameworks and may influence therapeutic decision-making, particularly as newer inhibitors and other targeted agents enter broader clinical use.

The historical classification of *NPM1*-mutated AML as a favorable-risk entity has long influenced clinical decisions, particularly when evaluating older or borderline-fit patients for intensive chemotherapy. Because *NPM1*-mutated disease often responds well to standard induction regimens [4], clinicians have sometimes leaned toward offering 7 + 3-based induction even when a patient’s physiological reserve was limited. This perception provides the rationale for the patient cohort examined in the recently presented PARADIGM trial that randomized patients to receive either 7 + 3- or CPX-351-intensive chemotherapy or azacitidine + venetoclax: in this study, patients with *NPM1*-mutated AML younger than 60 years were excluded [58]. However, determining the optimal intensive regimen remains controversial, especially amid evolving intensification strategies.

One major question has been whether adding gemtuzumab ozogamicin (GO) to intensive chemotherapy improves outcomes for *NPM1*-mutated AML. The German AMLSG 09-09 trial [59] found that the addition of GO reduced relapse rates and improved event-free survival, but did not improve overall survival, likely due to toxicity and early mortality from the intense chemotherapy regimen used for double induction and consolidation. Only 17% of AMSLG 09-09 patients were older adults, limiting generalizability to our patient population of interest.

The British analysis combining outcomes from the AML17 and AML19 trials provided additional insights [60]. Patients were randomized to four arms: daunorubicin/cytarabine versus FLAG-Ida, each with or without GO. Both FLAG-Ida and GO increased the proportion of patients achieving MRD negativity after cycle 2—a strong predictor of survival. FLAG-Ida also reduced relapse risk compared with 7 + 3. However, these intensified regimens were less suitable for older adults, and therefore only 20% of trial participants were over age 60.

Consequently, for most older adults with *NPM1*-mutated AML, azacitidine + venetoclax remains the preferred frontline approach. When examining the effects of this combination on outcomes, while the widely adopted ELN 2024 risk classification system (see above, Section 2.1.7) did associate good risk with the obligatory presence of specific mutations (*NPM1*, *IDH1/2*, *DDX41*), true risk in each of these mutational categories was actually defined by the absence of co-mutations associated with intermediate or poor risk (*FLT3*-ITD, *KRAS*/*NRAS*, *TP53*) [16]. Within this context, the authors demonstrated that when *NPM1* mutations did not occur with any of the adverse- or intermediate-risk mutations, the median overall survival was 27 months. This result is consistent with large multinational datasets showing that *NPM1* mutations are common among older AML patients and that outcomes vary with co-mutations [51].

Lachowiez et al. underscored the above (see above, Section 2.1.8) by reporting mutations that conferred good risk with azacitidine + venetoclax [23]—*NPM1*, *IDH1/2* and *DDX41*. Patients with one of these mutations did particularly well with azacitidine + venetoclax. This observation highlights that venetoclax-based therapy is an excellent treatment option for some molecular subsets. How the presence of two co-mutations that are both considered good risk may affect long-term responses remains an interesting question, as discussed below.

As discussed in Section 2.2), monocytic differentiation represents an important modifying factor related to reduced venetoclax sensitivity. Using unsupervised clustering of immunophenotype that identified four core markers, investigators confirmed that monocytic AML has inferior outcomes with azacitidine + venetoclax, especially within ELN-intermediate groups harboring *FLT3*-ITD, *RAS*, or *PTPN11* mutations [29]. Conversely, in *NPM1*-mutated AML, the opposite result was found: monocytic differentiation correlates with better outcomes, likely due to *NPM1*-driven upregulation of BCL2, restoring venetoclax sensitivity. It remains unclear which signaling pathway is activated by the *NPM1* mutation that is responsible for shifting dependence from one antiapoptotic protein to another, resulting in improved clinical outcomes [25].

The intriguing British/Australian results reported by Othman et al. using deep-MRD analyses have further refined our understanding of response quality in *NPM1*-mutated AML treated with azacitidine + venetoclax [52]. Using their ultra-sensitive *NPM1* quantitative PCR assay—which detects mutated transcripts at levels well below the “standard” flow cytometric MRD threshold of 10^−4^—they demonstrated that approximately 40% of patients treated with azacitidine + venetoclax can achieve profound molecular clearance by cycle 4 or 5. Such depth of remission is striking for a less-intensive regimen and correlates strongly with long-term outcomes. Patients who achieved deep MRD negativity experienced remarkably low relapse rates of approximately 10% and had substantially prolonged survival compared with those who remained MRD positive (whose relapse rates exceeded 70%). These findings reinforce the central role of MRD as a surrogate marker of therapeutic success in *NPM1*-mutated AML, and suggest that MRD kinetics may someday guide treatment duration, modifications, or sequencing strategies.

Co-mutational profiling revealed that patients harboring both *IDH1* and *NPM1* mutations achieved the highest MRD negativity rates in the azacitidine + venetoclax cohort [52]. This biologically favorable combination appears particularly sensitive to BCL2 inhibition, possibly due to complementary apoptotic and differentiation mechanisms activated by the two-drug combination. This observation raises an important clinical question: as both azacitidine + venetoclax and azacitidine + ivosidenib are viable options for patients with *IDH1*-mutated disease, how should clinicians select the optimal regimen for *IDH1*-mutated AML that carries an *NPM1* mutation? The number of patients with co-occurring *IDH1* and *NPM1* mutations in the AGILE study was quite small, limiting the ability to draw definitive conclusions about outcomes within this subgroup. Nonetheless, the British/Australian preliminary observations suggest that certain patients with overlapping mutations may have access to more than one effective therapeutic option. This conclusion is particularly encouraging in 2026, as it reflects a growing capacity to tailor therapy based on the biological underpinnings of each patient’s disease.

Another major therapeutic development for *NPM1*-mutated AML is menin inhibition. Mechanistic studies have established that mutant *NPM1* depends on the KMT2A (MLL)-menin epigenetic complex to maintain the aberrant HOX/MEIS expression that drives leukemogenesis [61,62]. Although NPM1 lacks enzymatic activity, it acts as a scaffold that stabilizes this transcriptional complex at HOX-enriched chromatin regions by bipartite, XPO-1-dependent binding to chromatin. Menin inhibitors disrupt this interaction, leading to the rapid downregulation of HOX/MEIS programs and the induction of differentiation, a mechanism shared by *NPM1*-mutated and *KMT2A*-rearranged leukemias.

Early clinical trials evaluating first-generation menin inhibitors, such as revumenib and ziftomenib, have demonstrated clinically meaningful activity, including molecular clearance and durable remissions in some patients [63,64]. Second-generation compounds including bleximinib and enzominib have entered clinical development. In phase 1/2 clinical trials, these inhibitors have all shown impressive monotherapy activity with CR rates of approximately 20% in relapsed/refractory patients, and CR rates > 90% in newly diagnosed patients when used in triplet combinations with azacitidine + venetoclax [65]. Multiple large international phase 3 studies are underway comparing such triplets to azacitidine + venetoclax in newly diagnosed patients, reflecting enthusiasm for this therapeutic strategy and the potential for synergy between BCL2 inhibition and epigenetic reprogramming (NCT06680453 (ziftomenib); NCT06852222 (bleximinib); NCT06652438 (revumenib)).

Furthermore, parallel trials for more fit patients assessing the addition of menin inhibitors to conventional chemotherapy (anthracycline and cytarabine) are planned or underway (e.g., KOMET-017, NCT07007312, HOVON 181/AMLSG 3721, NCT07223814). If successful, these combinations may fundamentally alter the therapeutic landscape of *NPM1*-mutated AML, much as IDH inhibitors transformed outcomes for *IDH*-mutated disease.

### 3.3. Summary

In summary, less-intensive therapy for favorable-risk AML is evolving rapidly. Oral decitabine/cedazuridine plus venetoclax may soon provide a fully oral option (see Section 3.1.2, *Venetoclax-based Regimens*, in the companion article in this issue of *Current Oncology,* entitled ‘Management of AML in Older Patients’). Azacitidine + ivosidenib offers a highly effective targeted regimen for *IDH1*-mutated AML. Intriguing analyses of subgroups indicate that venetoclax-based therapy may be especially potent for *NPM1*-mutated AML, with MRD-guided assessment and emerging triplet strategies offering further refinement. However, further follow-up to assess long-term durability of these responses, confirmation by other investigators, and comparison of outcomes from ongoing ivosidenib trials on the depth and duration of response for *IDH1-NPM1* co-mutated AML will help in treatment decisions. Looking forward, menin inhibitors represent one of the most promising advances in AML therapy today. If results from Phase III registration trials confirm the excellent results reported from the Phase I/II trials, the treatment landscape for treating *NPM1* mutated AML may be altered significantly in the not-too-distant future.

## 4. Treatment of Older AML Patients with Adverse Risk Mutations: FLT3-ITD and TP53

### 4.1. FLT3-Mutated AML

*FLT3* mutations are the most common genetic alterations in AML, identified in approximately one-third of newly diagnosed adults. *FLT3* internal tandem duplications (ITD) are found in about 25% of patients; point mutations in the tyrosine kinase domain (TKD) are found in about 5% [66,67]. The prevalence of *FLT3*-ITD mutations decreases slightly with age. However, as approximately 80% of AML cases occur in patients ≥ 60 years of age [66,68], a significant proportion of *FLT3*-mutated AML occurs in older adults.

The prognostic impact of *FLT3* mutations has evolved over time and also varies with treatment intensity. In intensively treated patients, *FLT3*-TKD mutations have generally been considered ‘neutral’ with respect to outcome, and such patients do not routinely proceed to alloSCT. In contrast, *FLT3*-ITD mutations have been associated with an inferior prognosis with intensive treatment, including high relapse rates and shorter overall survival [66,67]. Notably, however, the defined prognostic impact of *FLT3*-ITD abnormalities has varied over time. In ELN 2017, risk conferred by *FLT3*-ITD was defined by the presence or absence of *NPM1* co-mutations and by *FLT3*-ITD allelic burden, in the absence of cytogenetic abnormalities conferring favorable or adverse risk, and could range from favorable to adverse [3]. In ELN 2022, in contrast, FLT3-ITD cases are defined as intermediate risk in the absence of cytogenetic or molecular abnormalities classified as favorable or adverse [4].

In general, the treatment goal of intensively treated *FLT3*-ITD bearing AML has been to achieve a CR and then to proceed to alloSCT. This approach is obviously problematic in older patients, who may be intolerant of intensive chemotherapy and may also be too old/unfit for alloSCT. As a result, many such older patients with *FLT3*-ITD AML are treated less intensively, and only a subset are able to proceed to alloSCT. Such patients have generally been treated with *azacitidine* + venetoclax. As discussed above, the mPRS, ELN 2024, and Refined ELN 2024 risk classifications all define *FLT3*-ITD as intermediate risk in the absence of other abnormalities conferring adverse risk [7,16,23]. The recent availability of targeted therapy for *FLT3*-mutated AML is increasing the number of less-intensive treatment options, although such targeted approaches are still in their infancy in this setting. To date, three *FLT3* inhibitors have been approved by Health Canada and in other jurisdictions: midostaurin and quizartinib in frontline intensive therapy, and gilteritinib for relapsed/refractory disease.

#### 4.1.1. Newly Diagnosed Older Patients Receiving Less-Intensive Therapy

In the VIALE-A trial, 19% of patients had either a *FLT3*-ITD or a *FLT3*-TKD mutation. A subset analysis suggested benefit in this group with azacitidine + venetoclax over azacitidine alone (HR for death 0.66 (0.35–1.26) [5].

A subsequent post hoc analysis of pooled data from an earlier phase 1b study of azacitidine + venetoclax and the VIALE-A study reported more detailed outcomes in this group of patients (see Table 12) [69]. Among patients treated with azacitidine + venetoclax (and grouping *FLT3*-ITD and -TKD patients together), composite complete remission (CR/CRi) was 67% and median OS was 12.5 months (95% CI: 7.3–19.2). Not surprisingly, the median OS of patients with *FLT3*-TKD was more than twice that of patients with *FLT3*-ITD mutations (19.2 months vs. 9.9 months).

Previous ELN genetic risk classification systems were based on data from younger patients who received intensive chemotherapy and thus do not discriminate well for patients treated with azacitidine + venetoclax (see above, Section 2.1.1*).* The newer mPRS/ELN 2024 risk stratification systems (described in detail above, Section 2) are particularly targeted to defining risk in less-intensively treated patients. In mPRS/ELN 2024, *FLT3*-ITD mutations are considered intermediate-risk in patients treated with azacitidine + venetoclax, in the absence of a *TP53* mutation [7,16].

MD Anderson Cancer Center reported a phase 1/2 study that evaluated the triplet combination of azacitidine, venetoclax and the FLT3-inhibitor gilteritinib in two cohorts of *FLT3*-mutated AML patients: R/R patients and newly diagnosed patients who were unfit for intensive chemotherapy [70]. In the frontline setting, the triplet produced a very high remission rate (96% CR/CRi). MRD was measured by flow cytometry and by *FLT3*-ITD PCR. MRD negativity rates are shown in Table 13.

In the newly diagnosed cohort, 43% were able to proceed to alloSCT, which is a high proportion in an unfit population, although outcomes were not improved. The 18-month OS and relapse-free survival rates were 72% and 71%, respectively; 18-month OS rates for patients with *FLT3*-TKD and *FLT3*-ITD mutations were 100% and 61%. A gilteritinib dose of 80 mg/day was chosen for the phase 2 portion of the study due to concerns around myelosuppression with the regimen.

The recently reported interim results of the phase 1/2 VICEROY study validate the triplet approach and support the efficacy of the azacitidine, venetoclax, and gilteritinib triplet in newly diagnosed patients with *FLT3*-mutated AML ineligible for intensive induction [71]. In this study, gilteritinib was combined with azacitidine and patients were randomized to two different doses of venetoclax, 200 mg/day or 400 mg/day. The study reported high CR/CRi rates of approximately 90% in both arms. The 12-month OS rate was 64% with venetoclax 200 mg/day and 77% with venetoclax 400 mg/day.

Overall, the triplet combination is highly active and could serve as an effective bridge to alloSCT for many patients, although currently azacitidine + venetoclax remains a standard of care for unfit and older patients not eligible for intensive induction. A randomized phase 2 study comparing the triplet regimen to azacitidine + venetoclax in *FLT3*-mutated AML is currently underway as part of the NCI MyeloMATCH platform [72]. The azacitidine + venetoclax + gilteritinib triplet is not presently approved in Canada.

#### 4.1.2. Newly Diagnosed Older Patients Receiving Intensive Chemotherapy

Intensive chemotherapy with cytarabine and anthracycline plus a *FLT3*-inhibitor is a treatment option for fit, older adults with *FLT3*-mutated AML. This approach, combined with alloSCT, has historically been the primary treatment option for patients with *FLT3*-ITD and remains a standard-of-care treatment for fit younger patients [4]. Two *FLT3* inhibitors, midostaurin and quizartinib, currently have approval in the upfront setting in the US and Canada.

The approval of midostaurin was based on a large randomized, double-blind, placebo-controlled phase 3 trial (RATIFY, CALGB 10603, NCT00651261). Patients received standard chemotherapy plus either midostaurin or placebo. Both OS and event-free survival were significantly longer in the midostaurin group. The benefit of midostaurin was consistent across all *FLT3* subtypes [73].

While RATIFY did not enroll patients over 60 years of age, the German AMLSG 16-10 study (NCT01477606) did. This single arm, open label phase 2 study evaluated midostaurin plus intensive chemotherapy in newly diagnosed *FLT3*-ITD positive AML patients. Results were compared with a historical cohort of patients treated on 5 prior AMSLG trials, as well as with patients treated on the placebo arm of RATIFY. Post hoc multivariate analyses showed that the addition of midostaurin led to significant improvement in outcomes in both younger and older patients with AML and *FLT3*-ITD [74].

Quizartinib is a potent, 2nd-generation oral inhibitor of *FLT3*-ITD. It has been studied in the frontline setting in a randomized, double-blind, placebo-controlled phase 3 trial (QuANTUM-First, NCT02668653) [75], which compared quizartinib and placebo in combination with chemotherapy in induction and consolidation, and as maintenance following chemotherapy or alloSCT. Patients had newly diagnosed *FLT3*-ITD-positive AML and ranged in age from 20 to 75 years (median 56 years); 40% of patients were ≥60 years of age. Results showed improved overall survival in patients receiving quizartinib: median OS was 31.9 vs. 15.1 months with placebo, HR 0.78 (95% CI 0.62–0.98, *p* = 0.032). In a post hoc analysis, the HR for OS for patients younger than 60 years was 0.68 (95% CI 0.049–0.095) compared to 0.91 (95% CI 0.66–1.26) in patients 60 or over, although the study was not designed to compare these two groups.

There are no head-to-head prospective studies comparing midostaurin and quizartinib, so it is unknown which is the more effective treatment. Some considerations when using these two therapies include:

- Midostaurin (a Type I inhibitor) is also effective for *FLT3*-TKD mutations, which is not the case for quizartinib (a Type II inhibitor).
- Midostaurin and quizartinib have unique toxicity profiles that might affect drug choice (quizartinib, for example, is associated with QTc prolongation).
- FDA and Health Canada approvals of quizartinib included use as maintenance, which has become an increasingly important treatment consideration for this group of patients. In Canada, quizartinib is approved and available for induction together with 7 + 3 chemotherapy, as well as for maintenance therapy post-consolidation chemotherapy and post-alloSCT, although provincial reimbursements are not yet formalized. Thus, it might be reasonable to use quizartinib upfront (rather than midostaurin) in patients potentially proceeding to alloSCT. Midostaurin is not approved for maintenance therapy in North America, although it is approved for this indication in Europe.
- Two ongoing studies—HOVON 156 (NCT04027309) and PrECOG 0905 (NCT03836209)—are comparing directly upfront midostaurin and gilteritinib used together with intensive chemotherapy for induction and consolidation (and for maintenance in the HOVON study). Preliminary results of the HOVON 156/PASHA trial were presented at EHA 2026 [76], but we are all awaiting details of the final publication.
- Important unresolved questions for older patients include:
- What is the optimal initial treatment for fit older patients who potentially are candidates for both intensive induction or less-intensive treatment?
- For patients eligible for alloSCT, would less-intensive treatment with an azacitidine + venetoclax-based approach impact rates of alloSCT or outcomes following alloSCT?

#### 4.1.3. Older Patients with R/R FLT3-Mutated AML

Both intensive and non-intensive approaches have been described for R/R *FLT3*-mutated AML. The ADMIRAL study (NCT02421939) randomized 371 patients with R/R AML bearing *FLT3* mutations (median age 62 (range 19.0–85.0); *FLT3*-ITD, 88.4%, *FLT3*-TKD, 8.4%, both, 1.9%) 2:1 to gilteritinib 120 mg daily (n = 246) vs. chemotherapy (n = 109) [76]. The latter included 68 patients receiving intensive chemotherapy (MEC, 28; FLAG-Ida, 40) and 41 receiving less-intensive therapy (LDAC, 16; azacitidine, 25). Patients responding to gilteritinib were allowed to continue drug as maintenance therapy, both with and without alloSCT.

Gilteritinib monotherapy was superior to both intensive and less-intensive salvage chemotherapy approaches (see Table 14), and patients receiving gilteritinib were more likely to proceed to alloSCT. Notably, patients in the gilteritinib arm proceeding to alloSCT were permitted to continue gilteritinib post-transplant if possible, and such continuing patients demonstrated improved outcomes (post-alloSCT gilteritinib resumption was not randomized, however).

Gilteritinib is approved in Canada for R/R FLT3-mutated AML, including post-alloSCT, and is a suitable option for older patients for whom salvage chemotherapy (both intensive and less-intensive) is not appropriate. Gilteritinib is the only drug currently approved for this indication in Canada.

#### 4.1.4. AlloSCT and Maintenance Therapy in Older Patients with FLT3-Mutated AML

AlloSCT can be a curative option for some fit older patients with *FLT3*-mutated AML, although in very elderly or frail comorbid patients, this is not an option. However, both intensively and non-intensively treated patients may be alloSCT candidates.

As mentioned above, in the AMLSG 16–10 study, the addition of midostaurin led to significant improvement in outcomes in both younger and older patients with *FLT3*-ITD-mutated AML receiving intensive chemotherapy [74] (see above, Section 4.1.2). In that study, transplantation was intended for all patients who achieved CR/CRi after induction. Overall, 45% of patients received alloSCT in first CR/CRi: 48% in the younger cohort (18–60 years, n = 312) and 38% in the older cohort (61–70 years, n = 128).

A recent analysis of data from the Center for International Blood & Marrow Transplant Research (CIBMTR) reported on outcomes in 3147 patients with *FLT3*-ITD mutated AML who underwent alloSCT [77]. In this study, 27% of patients were 60–69 years of age and 6% were ≥70 years of age. In patients who underwent alloSCT in first CR, 4-year overall survival, leukemia-free survival, relapse after alloSCT, and non-relapse mortality were 55.5%, 50%, 33.7% and 16.3%, respectively. Leukemia-free survival was worse with increasing age in multivariate analysis, although this trend was observed in all patients 40 years and older.

Use of reduced intensity conditioning (RIC) in the CIBMTR study [77] appeared to be associated with higher relapse risk and lower survival; this is consistent with a prospective Blood and Marrow Transplant Clinical Trials Network (BMT CTN) study that compared RIC to myeloablative conditioning in AML and myelodysplastic syndrome (MDS) [78]. Intensification of conditioning appears to be particularly beneficial for patients with detectable MRD around the time of alloSCT [79,80], although this strategy may not be feasible for many older patients.

The CIBMTR study also demonstrated increasing use of maintenance therapy post-transplant from 2014 to 2019 [77], and there is increasing evidence to support this strategy in *FLT3*-mutated AML. Two randomized controlled trials that used sorafenib maintenance for patients with *FLT3*-ITD mutated AML following alloSCT reported reductions in relapse risk and improved overall survival. Sorafenib is not currently marketed for AML and access is challenging in many areas, including Canada [81,82].

The QuANTUM-First study also included a maintenance phase (quizartinib or placebo), both post-chemotherapy and post-alloSCT [75] (see also Section 4.1.2). There was no randomisation at the maintenance phase, so it is difficult to determine the impact of this strategy on treatment efficacy. Post hoc analysis suggested that maintenance with quizartinib prevented relapse or death compared to placebo, although this benefit was most evident in patients who did not undergo alloSCT [83].

The MORPHO study (BMT CTN 1506, NCT02997202) was a randomized controlled trial of gilteritinib (a Type I inhibitor) compared to placebo given for up to 2 years following alloSCT for *FLT3*-ITD AML [84]. The study did not meet the primary endpoint for improvement in relapse-free survival (*p* = 0.0518). However, a pre-specified secondary analysis showed that gilteritinib improved relapse-free survival in patients with positive MRD (even low level) measured pre- or post-alloSCT (HR, 0.515 (95% CI, 0.316 to 0.838); *p* = 0.0065). The public funding of gilteritinib in the post-transplant setting is currently under review by Health Canada.

As described above (see Section 4.1.3), the ADMIRAL study (NCT02421939) randomized 371 patients with relapsed or refractory AML bearing *FLT3* mutations 2:1 to gilteritinib vs. chemotherapy [76]. Gilteritinib monotherapy was superior to both intensive and less-intensive salvage approaches both for initial response and for survival. Patients responding to gilteritinib were allowed to continue drug as maintenance therapy, both with and without alloSCT.

Gilteritinib is approved in Canada for R/R *FLT3*-mutated AML, including post-alloSCT, and is a suitable option for older patients for whom salvage chemotherapy (both intensive and less-intensive) is not appropriate. Gilteritinib is the only drug currently approved in Canada for this indication.

In the absence of *FLT3* mutation-specific maintenance therapy, intensively treated patients not proceeding to alloSCT would be candidates for maintenance therapy with oral azacitidine as defined in the Quazar study [85]. Oral azacitidine maintenance is not approved for less-intensively treated patients or for the post-alloSCT setting.

Taken together, the studies described in Section 4.1.3 and Section 4.1.4. suggest that patients may benefit from maintenance therapy, including in the post alloSCT setting, and that this approach should be pursued. Maintenance post-alloSCT is becoming a standard of care in many centers, although the most effective strategy remains unknown at present. Future studies will hopefully compare different forms of maintenance and will further use MRD-driven designs to determine the optimal approach in this setting.

### 4.2. TP53-Mutated AML in Older Patients

*TP53* mutations are found in 5–15% of AML patients overall (including 5–10% of de novo AML), with higher incidences in patients ≥ age 60, sAML/tAML (25–40%), adult pure erythroid leukemia, and cases with complex karyotype [7,86,87,88,89]. With unique features and outcome, *TP53*-mutated AML is now classified as a separate adverse-risk AML subtype in the 2022 ELN recommendations and the 2022 International Consensus Classification of Myeloid Neoplasms and Acute Leukemias (ICC 2022) [4,90], with dismal outcomes in currently available therapies [55,86]. While, in older patients, *TP53* mutations are associated with other high-risk factors such as complex cytogenetics and higher rates of adverse-risk co-mutations, by multivariate analysis, one study concluded that in patients with AML > 60 years of age, *TP53* mutations conferred the single greatest prognostic risk compared to other covariates [91]. Consistent with this, treatment outcomes are extremely poor.

In a meta-analysis of 12 studies that met detailed eligibility criteria defined according to the Population, Interventions, Comparisons, Outcomes and Study (PICOS) design, Daver et al. compared outcomes in patients with *TP53*-mutated AML treated with HMA, HMA + venetoclax, or intensive chemotherapy [92]. Results (see Table 15) show that over 50% of patients with *TP53*-mutated AML do not achieve CR, and median OS is only 6–6.5 months, regardless of the treatment approach.

#### 4.2.1. Prognosis of TP53-Mutated AML

The prognosis of AML patients with *TP53* mutations seems to correlate with the allelic burden. In monoallelic *TP53* mutations, the wild-type allele is preserved, while in biallelic (multi-hit) mutations, the second *TP53* allele is inactivated by mechanisms including point mutation, loss of heterozygosity (LOH), or copy-neutral LOH (cnLOH). ICC 2022 defines multi-hit status as the presence of ≥2 distinct *TP53* mutations (variant allele frequency (VAF) of each mutation ≥ 10%), or a single *TP53* mutation associated with either (a) deletion of the *TP53* locus at 17p13.1 evident on cytogenetic analysis, (b) a VAF ≥ 50%, or (c) cnLOH at the 17p *TP53* locus [90]. In the absence of comprehensive copy-number analysis, the presence of a complex karyotype is considered a multi-hit equivalent. In contrast, a single hit is defined as one *TP53* mutation with VAF ≥ 10% to <50% without LOH or cnLOH at the 17p13.1 locus [90]. Overall, approximately 70% of *TP53* mutated AML cases are biallelic [93].

Biallelic mutations are believed to confer a worse prognosis than do monoallelic mutations [93,94]. While this relationship has been shown clearly in MDS, and it has also been reported in AML in some studies, it remains debated [88]. Notably, in one study, median OS of AML patients with biallelic *TP53* mutations was only 1 month [93]. A recent study including AML patients suggested a lower VAF cut-off (>23%) may more accurately identify patients with biallelic mutations and worse prognosis [95].

#### 4.2.2. Induction Therapy

The overall survival of patients with *TP53*-mutated AML who are treated with less-intensive HMA-based induction therapy is comparable to that of patients treated with intensive induction therapy (see Table 15). It seems reasonable to suggest, therefore, that the majority of older patients with *TP53* mutations be treated with HMA-based therapy [92]. If available, clinical trials should be prioritized for this subset of AML patients.

A post hoc analysis of the VIALE-A study showed that patients with *TP53* mutations had a much higher response rate (CR/CRi 55% vs. 0%) with azacitidine + venetoclax than with azacitidine + placebo. However, there was no difference in OS, which was approximately 5 months in both groups [5,7]. It has been suggested, therefore, that HMA + venetoclax should be reserved for patients designated for alloSCT. If alloSCT is planned for a *TP53*-mutated AML patient, treatment with HMA + venetoclax may be appropriate to reduce blast count and leukemic burden, with the patient proceeding promptly thereafter to alloSCT. By extension, therefore, for more frail/older patients not eligible for alloSCT, HMA alone may be preferred as it can offer a similar outcome with fewer side effects. An alternative viewpoint argues, however, that the quality-of-life advantages derived from achieving a CR (transfusion independence, fewer infections, fewer hospital visits, etc.) may favor HMA + venetoclax over HMA alone for a wider range of patients, even if such advantages are not durable [96].

#### 4.2.3. Transplantation

Decision-making regarding alloSCT in patients with *TP53*-mutated AML is challenging. This procedure may improve survival and be curative in a minority of patients, but the vast majority will relapse following transplantation. The procedure is also resource-intensive and can result in significant morbidity and reduced quality of life. Notably, longer-term OS following alloSCT has been reported as <20% in most series [97], although a recent retrospective multicenter study suggested slightly better outcomes in this group [98]. It should also be noted that factors such as conditioning intensity and donor choice do not seem to impact outcomes significantly [99].

Based on the available evidence, we suggest that in older patients, factors such as suspected monoallelic disease, CR/CRi prior to alloSCT, better performance status, and low hematopoietic cell transplant-comorbidity index (HCT-CI) may be associated with better post-alloSCT outcomes and could be used to help select patients for this procedure (see Table 16). It is important to be transparent with patients planning for alloSCT regarding the uncertainty of benefit from the procedure and the high risk of relapse post-transplant.

While much of the AML *TP53* literature remains controversial, especially in the elderly and regarding alloSCT, there is general agreement that there remains a large unmet need for novel treatments in *TP53*-mutated AML. Not only are outcomes poor with standard approaches, both intensive and less intensive, but clinical trials to date have failed to yield breakthrough therapies. Approaches in *TP53*-mutated AML have included, among others [88,89], (a) modulation of TP53 function by epigenetic targeting (azacitidine and decitabine ± venetoclax), restoration of TP53 function (eprenetapopt (APR-246), arsenic trioxide), and degradation of mutant TP53 (statins); (b) targeting dependent molecular pathways to overcome intrinsic drug resistance (BCL2 and MCL1 inhibition, entrectinib); and (c) the recruitment of immune responses via immunotherapy (magrolimab, sabatolimab, flotetuzumab, nivolumab) or via cellular therapies (various CAR-T approaches). It is essential that, going forward, such patients should be enrolled in clinical trials.

## 5. Conclusions and Future Directions

While the topics discussed in this article may appear unconnected, they are in fact interrelated. Less-intensive AML treatment has evolved over the last decade in parallel with an improved understanding of AML disease biology. The latter has facilitated better genetic and phenotypic disease stratification, as well as an increasing understanding of the interrelatedness between single and combinatorial genetic abnormalities and phenotype, particularly with respect to regulation of apoptosis. By extension, this has led reciprocally to a further refinement in our understanding of risk. For example, *NPM1*-mutated AMLs comprise several subgroups with distinct risk phenotypes that may define specific treatment approaches. Refined classification also confirms the ‘bad players’. *FLT3*-mutated AML remains problematic, although it is anticipated that new drug approvals and MRD-based treatment approaches may alleviate this, at least in part. And, in particular, *TP53*-mutated AML remains a major problem. Ongoing clinical trial enrollment is essential.

## Figures and Tables

**Table 1:** VIALE-A: Responses to azacitidine + venetoclax vs. azacitidine + placebo in patients with untreated AML ineligible for intensive chemotherapy due to age ≥ 75 or comorbidities [5].

	AZA + VEN	AZA + PBO	
CR	36.7%	17.9%	*p* < 0.001
CR/CRi	66.4%	28.3%	*p* < 0.001
Median OS	14.7 mos	9.6 mos	HR 0.66 *p* < 0.001

AML, acute myeloid leukemia; AZA, azacitidine; CR, complete remission; CRi, complete remission with incomplete hematologic recovery; HR, hazard ratio; mos, months; OS, overall survival; PBO, placebo; VEN, venetoclax.

**Table 2:** VIALE-A and phase 1b study (pooled analysis): response and overall survival according to ELN 2017 and ELN 2022 risk categories [7].

	ELN 2017		ELN 2022	
	AZA + VEN	AZA + PBO	AZA + VEN	AZA + PBO
CR/CRi				
Favorable risk	69.6%	20.0%	74.3%	15.4%
Intermediate risk	75.4%	61.3%	70.7%	46.7%
Adverse risk	61.3%	25.7%	63.5%	25.9%
Median OS				
Favorable risk	21.1 mos	13.0 mos	39.0 mos	11.0 mos
Intermediate risk	23.3 mos	13.1 mos	15.2 mos	9.1 mos
Adverse risk	11.5 mos	7.4 mos	12.7 mos	9.3 mos

AZA, azacitidine; CR, complete remission; CRi, complete remission with incomplete hematologic recovery; ELN, European LeukemiaNet; OS, overall survival; PBO, placebo; VEN, venetoclax.

**Table 3:** mPRS risk model based on the mutational status of four genes [7].

mPRS Group	Mutational Status
Higher benefit	*TP53*^wt^, no *FLT3*-ITD, *K/NRAS*^wt^
Intermediate benefit	*TP53*^wt^, *FLT3*-ITD and/or *K/NRAS*^mut^
Lower benefit	*TP53* ^mut^

mPRS, Molecular Prognostic Risk Signature; mut, mutated; wt, wild type.

**Table 4:** Mayo Clinic genetic risk model [9].

A. Genetic Features	Points	** **	B. Risk Groups	Points	Median OS	3-yr OS
ELN 2022 adverse risk karyotype	1		Low	0	NR	67%
*IDH2* ^wt^	1		Intermediate	1	19.1 mos	33%
*TP53* ^mut^	1		High	≥2	7.1 mos	0%
*KRAS* ^mut^	1					
*KMT2Ar*	2					

ELN, European LeukemiaNet; mos, months; mut, mutant; NR, not reported; OS, overall survival; wt, wild type; yr, year.

**Table 5:** AGILE study: azacitidine + ivosidenib vs. azacitidine + placebo in patients with *IDH1*-mutated untreated AML ineligible for intensive chemotherapy [11,13].

	AZA + IVO	AZA + PBO	
Response rates			
CR	47%	15%	
CR/CRi	53%	18%	
ORR	63%	19%	
Median OS			
Planned primary analysis (median follow-up 12 mos)	24.0 mos	7.9 mos	HR 0.44 *p* = 0.001
Unplanned post hoc analysis (median follow-up 28.6 mos)	29.3 mos	7.9 mos	*p* < 0.0001

AML, acute myeloid leukemia; AZA, azacitidine; CR, complete remission; CRi, complete remission with incomplete hematologic recovery; HR, hazard ratio; IVO, ivosidenib; mos, months; ORR, overall response rate; OS, overall survival; PBO, placebo.

**Table 6:** ELN 2024 genetic risk classification of HMA-naïve patients (adapted from [16]).

Risk Categories	Mutational Status	Median OS (mos)	Refs
Favorable ^a^	Mutated *NPM1* (*FLT3*-ITD^neg^_,_ *NRAS*^wt^, *KRAS*^wt^, *TP53*^wt^)	39	[7]
	Mutated *IDH2* (*FLT3*-ITD^neg^_,_ *NRAS*^wt^, *KRAS*^wt^, *TP53*^wt^)	37	[11,17]
	Mutated *IDH1* ^a^ (*TP53*^wt^)	29	[7]
	Mutated *DDX41* ^b^	>24	[14,18]
	Other cytogenetic abnormalities and/or molecular abnormalities ^c,d^ (*FLT3*-ITD^neg^_,_ *NRAS*^wt^, *KRAS*^wt^, *TP53*^wt^)	23	[7]
Intermediate	AML with MR gene mutations ^d^	13	[7]
	Other cytogenetic and/or molecular abnormalities (*FLT3*-ITD^pos^ and/or *NRAS*^mut^ and/or *KRAS*^mut^; *TP53*^wt^)	12	[7]
Adverse	Mutated *TP53*	5–8	[7,8,14,19,20,21,22]

a Only applies to patients receiving AZO + IVO, irrespective of the presence of activating signaling gene mutations. b Identification of a DDX41 mutation at near-heterozygous frequency should prompt consideration of a germline DDX41 mutation. c Includes AML with MR gene mutations. d For many cytogenetic and molecular abnormalities, single or as co-aberrations, no data are currently available; they are tentatively categorized as favorable and intermediate-risk depending on the absence or presence of activating signaling gene mutations. mos, months; MR, myelodysplasia-related; mut, mutated; neg, negative; OS, overall survival; pos, positive; Refs, references; wt, wild type.

**Table 7:** Median OS of patients treated with HMA + venetoclax, by ELN 2024 risk category and specific mutational status [23].

ELN 2024 Risk Category	Mutational Status	Median OS (mos)	
Favorable	*NPM1^mut^, IDH1/2^mut^, DDX41^mut^*	34.8	*p* = 0.0006
	*NPM1^wt^, IDH1/2^wt^, DDX41^wt^*	8.6	
Intermediate	*KRAS^mut^*	3.3	*p* = 0.016
	*KRAS^wt^*	13	
Intermediate	*NRAS^mut^*	8.6	*p* = 0.059
	*NRAS^wt^*	15.4	
Favorable/ intermediate	*PTPN11^mut^*	4.9	*p* = 0.005
	*PTPN11^wt^*	16.2	

ELN, European LeukemiaNet; HMA, hypomethylating agent; mos, months; mut, mutated; OS, overall survival; wt, wild type.

**Table 8:** Median OS by risk category, comparing ELN 2024 and refined ELN 2024 risk stratification systems [23].

Risk Category	Median OS (mos)	
	ELN 2024	Refined ELN 2024
Favorable	17.2	34.8
Intermediate	10.2	13.0
Adverse	6.5	5.4

ELN, European LeukemiaNet; mos, months; OS, overall survival.

**Table 9:** PRISM score: variables included (listed in order of decreasing weight) [24].

More Favorable	More Adverse
Missing karyotype	*KRAS* ^mut^
*BCOR* ^mut^	*TP53* ^mut^
*IDH1* ^mut^	ELN 2022 adverse karyotype
*STAG2* ^mut^	sAML
*RUNX1* ^mut^	*FLT3*-ITD
*IDH2* ^mut^	*JAK2* ^mut^
*CEBPA*^mut^ (both bZIP + non-bZIP)	*ASXL1* ^mut^
	ELN 2022 intermediate karyotype
	Sex (male)
	Age

ELN, European LeukemiaNet; mut, mutated; PRISM, Prognostic Risk Integration for Survival Modeling; sAML, secondary AML.

**Table 10:** Redistribution of mPRS-classified patients by PRISM score [24].

Risk Category	% Re-Classified by	% Re-Classified to		
by mPRS	PRISM Score	Low Risk	Moderate Risk	High Risk
Favorable	52.5		41.3	11.2
Intermediate	59.1	30.2		28.9
Adverse	6.1	0.3	5.8	

mPRS, Molecular Prognostic Risk Signature; PRISM, Prognostic Risk Integration for Survival Modeling.

**Table 11:** Median OS: PRISM score vs. mPRS [24].

	Median OS ^a^ (mos)	
Risk Category	mPRS	PRISM Score
Favorable	17.6	23.8
Intermediate	11.4	14.6
Adverse	6.9	6.6

a Estimated from abstract presentation. mos, months; mPRS, Molecular Prognostic Risk Signature; OS, overall survival; PRISM, Prognostic Risk Integration for Survival Modeling.

**Table 12:** Remission rates and overall survival in patients with *FLT3*-mutated AML treated with azacitidine + venetoclax (post hoc pooled analysis of phase 1b study of azacitidine + venetoclax and VIALE-A) [69].

	*FLT3^wt^*	*FLT3^mut^* *	*FLT3*-ITD	*FLT3*-TKD
CR	40%	38%	30%	54%
CR/CRi	67%	67%	63%	77%
CR/CRi duration Median (95% CI)	18.4 mos (15.1-NE)	17.3 mos (10.1-NE)	17.3 mos (4.6-NE)	15.9 mos (28-NE)
Median OS by therapy				
AZA + PBO	10.1 mos	8.6 mos		
AZA + VEN	14.7 mos	12.5 mos	9.9 mos	19.2 mos

* *FLT3*^mut^, *FLT3*-ITD and -TKD combined. AML, acute myeloid leukemia; AZA, azacitidine; CR, complete remission; CRi, complete remission with incomplete hematologic recovery; mos, months; mut, mutant; NE, not evaluable; PBO, placebo; VEN, venetoclax; wt, wild type.

**Table 13:** MRD negativity rates with triplet therapy (azacitidine, venetoclax and gilteritinib) in newly diagnosed and R/R *FLT3*-mutated AML patients [70].

	Newly Diagnosed (n = 30)	R/R (n = 22)
MRD after cycle 1		
by flow cytometry	56%	11%
by PCR for *FLT3*	37%	27%
MRD: best response		
by flow cytometry	93%	45%
by PCR for *FLT3*	90%	43%

AML, acute myeloid leukemia; MRD, measurable residual disease; PCR, polymerase chain reaction; R/R, relapsed/refractory.

**Table 14:** Remission rates and overall survival in patients with R/R *FLT3*-mutated AML treated with gilteritinib or chemotherapy [76].

	Gilteritinib	Chemotherapy
CR	21.1%	10.5%
CCR *	54.3%	21.8%
Median EFS	2.8 mos	0.7 mos
Median OS	9.3 mos	5.6 mos
alloSCT	25.5%	15.3%

* Composite complete remission was defined as the combination of complete remission, complete remission with incomplete hematologic recovery, and complete remission with incomplete platelet recovery. AML, acute myeloid leukemia; CCR, composite complete remission; CR, complete remission; EFS, event-free survival; mos, months; OS, overall survival.

**Table 15:** Responses and outcomes in *TP53*-mutated AML by treatment approach [92].

	IC	HMA + VEN	VEN
CR	43%	33%	13%
CR/CRi	46%	49%	13%
ORR	41%	65%	47%
OS	6.5 mos	6.2 mos	6.1 mos

AML, acute myeloid leukemia; CR, complete remission; CRi, CR with incomplete hematologic recovery; HMA, hypomethylating agent; IC, intensive chemotherapy; mos, months; ORR, overall response rate; OS, overall survival; VEN, venetoclax.

**Table 16:** Proposed recommendations for alloSCT in patients with *TP53*-mutated AML (adapted from [97]).

**Disease:**	Monoallelic TP53 mutation without complex karyotype	Biallelic TP53 loss and/or complex karyotype	
**Patient:**	Fit for alloSCT	KPS ≥ 90; HCT-CI < 4 ^a^	KPS < 90; HCT-CI ≥ 4 ^a^
**Offer** **alloSCT?**	Yes	Maybe	No
**Comments:**	Apply standard fitness criteria	Donor options may modify risk assessment (e.g., alloSCT may be less viable in older patients if only alternative donors are available). Must clearly discuss risks, benefits and expectations regarding post-transplant prognosis.	Consider referral for second opinion regarding alloSCT.

a Score intended as provisional guidance, not as an absolute threshold. Consider alternative non-relapse mortality scoring systems [100,101]. alloSCT, allogeneic stem cell transplant; HCT-CI, hematopoietic cell transplant-comorbidity index; KPS, Karnofsky performance status.

## Data Availability

No new data were created or analyzed in this study. Data sharing is not applicable to this article.

## Abbreviations

α-KG	α-ketoglutarate
2-HG	2-hydroxyglutarate
alloSCT	allogeneic stem cell transplantation
AML	acute myeloid leukemia
AMLSG	Acute Myeloid Leukemia Study Group
APL	acute promyelocytic leukemia
ATRA	all-trans retinoic acid
BCL-2	B-cell lymphoma 2
BMT CTN	Blood and Marrow Transplant Clinical Trials Network
CDA	Canadian Drug Association
CIBMTR	Center for International Blood & Marrow Transplant Research
CLSG/GCEL	Canadian Leukemia Study Group/Groupe canadien d’étude sur la leucémie
CMML	chronic myelomonocytic leukemia
cnLOH	copy neutral loss of heterozygosity
CR	complete remission
CCR	composite complete remission (CR/CRi)
CRi	complete remission with incomplete hematologic recovery
DEC-C	oral decitabine and cedazuridine
DS	differentiation syndrome
EHA	European Hematology Association
ELN	European LeukemiaNet
EMA	European Medicines Agency
FDA	U.S. Food and Drug Administration
FLAG-Ida	fludarabine, cytarabine, granulocyte colony-stimulating factor and idarubicin
*FLT3*	fms-like tyrosine kinase 3
*FLT3*-ITD	*FLT3*-internal tandem duplication
*FLT3*-TKD	*FLT3*-tyrosine kinase domain
GO	gemtuzumab ozogamicin
HCT-CI	hematopoietic cell transplant-comorbidity index
HMA	hypomethylating agent
HR	hazard ratio
IC	intensive chemotherapy
ICC	International Consensus Classification of Myeloid Neoplasms and Acute Leukemias
IDH	isocitrate dehydrogenase
ITD	internal tandem duplication
LDAC	low-dose cytarabine
LOH	loss of heterozygosity
LSC	leukemic stem cell
MAC score	mediators of apoptosis combinatorial score
MCL-1	myeloid cell leukemia-1
MDACC	MD Anderson Cancer Center
MDS	myelodysplastic syndrome
MPN	myeloproliferative neoplasm
mPRS	Molecular Prognostic Risk Signature
MRC	Medical Research Council (UK)
MRD	measurable residual disease
mut	mutated
neg	negative
NGS	next generation sequencing
NOS	not otherwise specified
*NPM-1*	nucleophosmin
OS	overall survival
PCR	polymerase chain reaction
POS	positive
PRISM	prognostic risk integration for survival modeling
RCT	randomized controlled trial
RIC	reduced intensity conditioning
R/R	relapsed/refractory
sAML	secondary AML
tAML	therapy-related AML
TKD	tyrosine kinase domain
TKI	tyrosine kinase inhibitor
VAF	variant allele frequency
wt	Wild type
